# Supplementary material for: Establishment of Canine Oral Mucosal Melanoma Cell Lines and Their Xenogeneic Animal Models
Source: Cells. 2024 Jun 6;13(11):992. doi: 10.3390/cells13110992 (PMC11171988; doi:10.3390/cells13110992)
Supplement: Supplementary file 1 [file cells-13-00992-s001.zip › cells-2966573-supplementary.pdf]

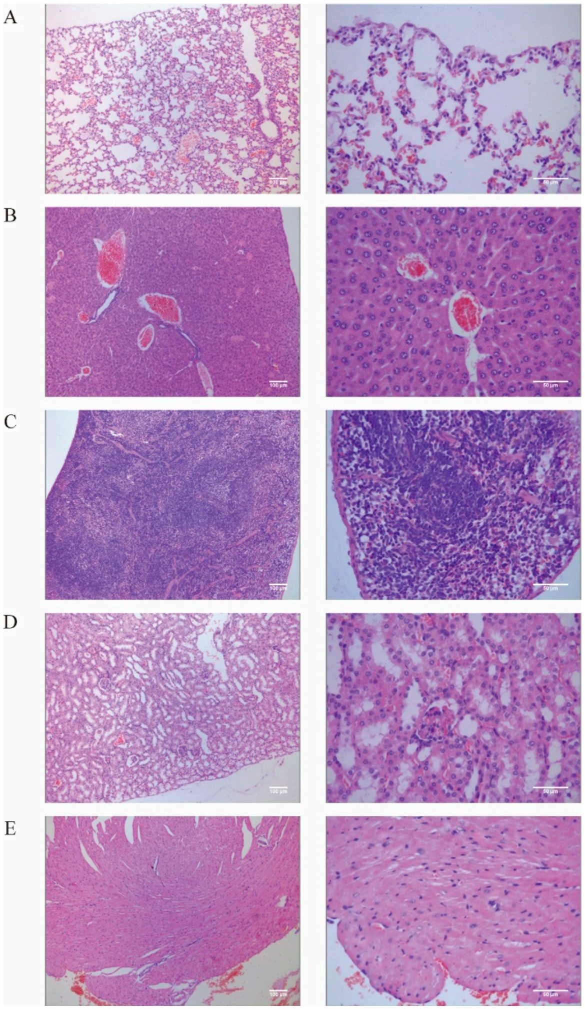

Figure. S1. Pathological sections show that none of the major organs of BALB/c-nu mice in the oral intra-lingual implant and subcutaneous implant trials developed tumor metastasis during the test cycle. **(A-E)** Pathologic tissue sections of lung, liver, spleen, kidney, and heart, in that order. Scale bar 100 μm(left column); scale bar 50 μm(right column).
